# Annotations

**Published:** 1898-07-09

**Authors:** 


### ANNOTATIONS.

### Medical Missionaries.

We are in full sympathy with those who would send out medical men as missionaries to the heathen; in fact we can see no better way of spreading among ignorant nations a knowledge of the better side of Christian civilisation than the plan of sending, as the scouts and advance guards of Christianity, missioners who are as skilled in ministering to the wants of the body as they are in setting forth the truths of the Gospel. Whether it is well, however, that young men should be trained from their youth up as medical missionaries, and whether it is wise that during their medical education men undergoing such training should receive pecuniary aid, are questions of great complexity which should not, we think, be decided hastily. The five years' curriculum is a large slice out of the most receptive period of a young man's life; and, while we cannot but feel thankful that, there is a prospect of a residential college, in which young men may pass what is always a trying and critical period amid good influences and under the glamour of those lofty aims which lead to self-sacrifice and to devotion to the service of others, we cannot forget that five years is a long time, that there are many probabilities of change of opinion during that time, and that any pecuniary obligation, by which young men may feel themselves tied down to a career their "call" for which may have faded and passed away before the time has arrived for them to seriously take it up, will not conduce to the selection of the best and most fitting candidates for missionary work. Youthful enthusiasm is no doubt prolific in the production of martyrs, but the best missionaries are those who take up the work after they have become mature. The worst probably are those who, having begun with enthusiasm, are disillusioned before their time for action arrives, and yet feel bound to persist in a career to which they feel that, in honour, they have tied themselves; and this is a stumbling-block which will have to be carefully guarded against in the schemes which are now being set on foot for forming colleges for the training, and especially for the pecuniary assistance, of medical missionaries.

### Philanthropists and the Workmen's Compensation Act.

Philanthropists, of whom there are many shades, will do well to consider carefully the provisions of the Workmen's Compensation Act. In times of distress it is not uncommon to see relief works of various kinds established, and although at first sight one might imagine that, the object being in such cases to provide an outlet for labour of a most unskilled character, the use of mechanical power would be subversive of their very aim, it has to be remembered that such relief works must be economically conducted or else their products cannot be sold without great loss. Hence we find that steam and other forms of mechanical power have not infrequently to be employed, and when this is done it will often bring the employés under the provisions of the Act. Ordinary road-making would probably be exempt, although even road-making might perhaps come within the definition of engineering work, especially if steam rollers were used, and thus might render a charitable association or a board of guardians liable. Setting men to dig in a quarry or to make a sewer would certainly qualify them for compensation, as also would the use of a steam saw in a wood-chopping shed. Laundry work also comes under the Act wherever steam power is employed. There can be little doubt that, thoroughly good as is the object of the Act, its influence on employment will be very widely felt in many unexpected directions. Already we hear of old men being discharged as being more liable to accident than their younger and more active fellows. On the other hand, we should expect that gradually, as the working of the Act gets more understood, its operations will put a high premium upon general steadiness of conduct, and unless the systems of insurance which will be introduced should be so worked as to make it easier for the master to insure and have done with it, rather than to trouble any further about the safety of his men, we should not be at all surprised to find that one of the effects of the Act will be to greatly encourage what may be called "pledged teetotalism"; for there can be little doubt that, whatever his power as a workman may be, the absolute teetotaler is less likely to meet with accident in the course of his work than those men are whose nerves are unsteadied by the use of alcohol.

### The Metropolitan Asylums Board.

At the meeting of the Metropolitan Asylums Board held last Saturday, Mr. Acworth presented the annual report of the Statistical Committee, with the appendices, containing the reports of the medical officers. Among matters of interest mentioned may be noted the fact that the proportion of admissions to the total number of legally admissible cases continues to rise, having in 1897 been 58,5 per cent., of all which were notified All scarlet cases which applied were admitted from the beginning of the year up to about the middle of July, the proportion of notified cases admitted during those months averaging 72·45 per cent. of the total. It is to be observed, however, that even this very considerable amount of isolation of infectious cases did not suffice to prevent the usual increase of the disease during the summer and autumn months, or to prevent it outrunning even the large amount of accommodation provided, so that again the managers have to record the entire failure of that accommodation to meet all the demands upon it during the season of greatest pressure. Much stress is laid on the steady diminution of the fatality among the cases treated in the hospitals of the Board, but that is a complex problem, regarding which the last word has probably not yet been said. We have no doubt that of recent years the fatality of scarlet fever, for example, has diminished; but there are large cycles in these matters, and the meaning of this diminution all depends upon whereabouts we happen to be just now on one of these great cyclical curves of fatality. The lessened fatality of diphtheria after the introduction of antitoxin is another matter, as in that case the fall was a sudden one, the comparison being made between two contiguous groups of years. The medical report we shall consider in a future article.

